# Design of a New Acoustic Logging While Drilling Tool

**DOI:** 10.3390/s21134385

**Published:** 2021-06-26

**Authors:** Kai Zhang, Baohai Tan, Wenxiu Zhang, Yuntao Sun, Jian Zheng, Yuanda Su, Xutang Liu, Gaofu Wu, Shoutao Xin

**Affiliations:** 1 School of Geosciences and Technology, China University of Petroleum, Qingdao 266555, China; tanbaohai@upc.edu.cn (B.T.); suyuanda@upc.edu.cn (Y.S.); 2 Institutions of Earth Science, Chinese Academy of Sciences, Beijing 100029, China; zhangwenxiu@mail.iggcas.ac.cn (W.Z.); sunyuntao@mail.iggcas.ac.cn (Y.S.); zhengjian@mail.iggcas.ac.cn (J.Z.); 3 College of Mechanical and Electrical Engineering, China University of Petroleum, Qingdao 266555, China; lxt220@upc.edu.cn; 4 East China Measurement and Control Branch of Sinopec Matrix Corporation, Yangzhou 225000, China; foxdy23@163.com; 5 ChangQing Branch of CNPC Logging, Xi’an 710075, China; xinshoutao@163.com

**Keywords:** acoustic logging while drilling, acoustic logging tool, sine wave pulse excitation, broadband impedance matching, intellectualized active reception transducer

## Abstract

To obtain qualified logging while drilling (LWD) data, a new acoustic LWD tool was designed. Its overall design is introduced here, including the physical construction, electronic structure, and operation flowchart. Thereafter, core technologies adopted in this tool are presented, such as dominant exciting wave bands of dipole source, a sine wave pulse excitation circuit, broadband impedance matching, and an intellectualized active reception transducer. Lastly, we tested this tool in the azimuthal anisotropy module well, calibration well, and normal well, working in the model of the cable, sliding eye, and logging while drilling. Experiments showed that the core technologies achieved ideal results and that the LWD tool obtained qualified data.

## 1. Introduction

Acoustic logging adopts transmitting transducers to transform electric signals into acoustic signals, which then propagate into the deep underground layer, and are finally collected by receiving transducers. These signals are stored and analyzed for oil and gas exploration and exploitation [1]. Traditional acoustic logging proceeds after drilling and cementing; however, acoustic logging while drilling (LWD) not only rapidly obtains traditional acoustic logging messages, but also predicts the reservoir pressure to improve drilling security, and obtains formation messages to guide the drilling orientation [2,3]. To date, acoustic LWD technology has progressed from testing formation longitudinal waves (the 1990s) to shear waves (the 2000s), and lastly to formation directional characteristics (2010) [4,5,6]. The most representative tools are SonicScope of the Schlumberger company [7], SoundTrak of the Baker Hughes company, XBAT from Halliburton, and Crosswave of the Weatherford company [8,9]. Although great success has been achieved with these tools, little technical documentation has been acquired as core technical documentation.

To obtain qualified LWD data, the crucial problem of the great noise confusion must be resolved. Compared with traditional wireline logging, acoustic LWD is confronted with much more noise confusion, such as drilling noise, mud circulation noise, and collar waves (excited by the excitation transducer and distributed along the collar) [10,11,12,13]. Besides the domain of oil and gas exploration, transducers play an important role in many other domains [14,15,16], such as the military, agriculture, and medicine. Additionally, there are many domains requiring measurement in the process of drilling [17,18], and which face the same problems. This paper contains several crucial technologies for improving the transmitting power and the signal-to-noise ratio, which are both of great significance to researchers.

To obtain powerful transmitting signals, we proposed three technologies: the dominant exciting wave bands of dipole source, the sine wave pulse excitation circuit method, and the broadband impedance matching technique. To improve the signal-to-noise ratio of the reception signals, we adopted an intellectualized active reception transducer, and thereafter, selected 32 reception transducers of obvious consistency. Based on the above technologies, a new acoustic LWD tool was designed and tested [19].

## 2. Overall Design of the Acoustic LWD Tool

**Figure 1:**
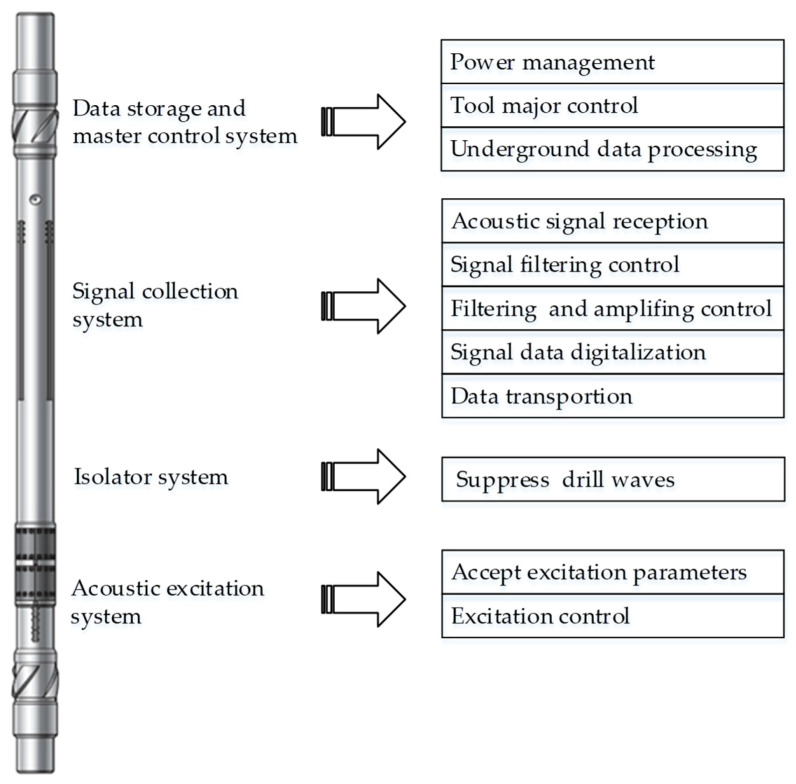
Schematic representation of the structure of the designed acoustic logging while drilling tool, and main functions of each part.

The designed LWD tool consists of a data storage and master control system, a signal collection system, an isolator system, and an acoustic signal excitation system (Figure 1).

The data storage and master control system is at the top of the LWD tool; it is for power management, major tool control, underground data rapid processing, and communication with surface devices.

The signal collection system consists of many receiver transducers and their processing circuits. First, this system accepts collection parameters from the master control system. Then, according to these parameters, it realizes acoustic signal reception and filtering, amplifier gain control, and signal data digitalization and processing. Lastly, these data are transported to the master control system, according to their address and based on programmed working frequencies.

The isolator is between the transformer transducers and receiver transducers, and is used to suppress the drill waves excited by the monopole and dipole sources.

The transducer excitation system is at the bottom of the tool. It contains four transformer transducers and transducer excitation electronic circuits. This system accepts excitation parameters from the master control system, then controls the frequency conversion excitation of high-power multipole acoustic signals, and lastly, emits acoustic signals into the underground layers.

**Figure 2:**
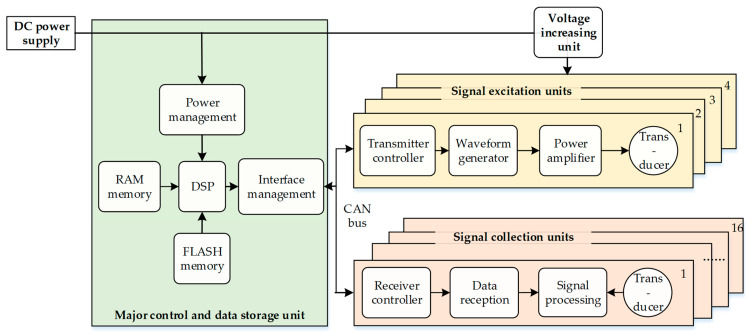
Structural diagram of the electronic system in the designed LWD tool.

Correspondingly, the LWD tool’s electronic system contains a major control and data storage unit, four acoustic signal excitation units, and sixteen signal collection units (Figure 2).

The major control and data storage unit contains a power management circuit, two memories (random access memory and flash memory), a digital signal processing processor, and an interface management circuit. The memories are used to place the LWD data. The processor is used to control the working status of the excitation unit and for management of the power supply. The interface management circuit adopts a controller area network (CAN) to realize order communication and data transport between these three units.

The signal excitation unit contains a transmitter controller, a waveform generator, a power amplifier, and an excitation transducer. The transmitter controller accepts transmitting parameter orders by CAN bus. The waveform generator realizes instant signals of adjustable frequencies, shapes, and voltages. The power amplifier changes these signals into high-voltage signals, and lastly, these signals are transmitted to monopole, dipole, and quadrupole transducers.

The reception unit contains a receiver controller, a data reception circuit, a signal processing circuit, and a reception transducer. The signal processing circuit receives signals from the reception transducers and processes programmed signal amplification and filtration. The data reception circuit changes analog signals into digital signals. Meanwhile, the receiver controller receives the receiving parameter orders by CAN bus and controls the whole process of data reception.

**Figure 3:**
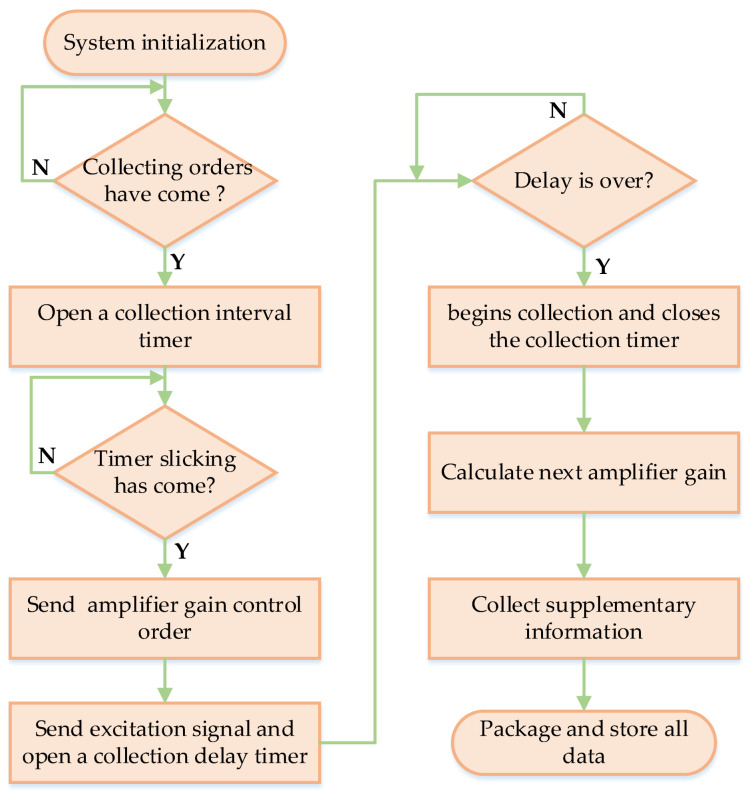
Flowchart of the designed tool working in logging pattern.

The working frequencies of this LWD tool working in LWD logging mode are shown in Figure 3. When electricity is supplied, this tool’s underground system performs system initialization, and can be set to sleeping status. Thereafter, it waits to collect orders after its master controller botting time is set by the surface device. When the collecting orders have arrived, the system will open a collection interval timer. After the timer slicking has arrived, the system sends the amplifier gain control order. Thereafter, it sends the control signal to the excitation system to excite the firing transducers; meanwhile, it opens a collection delay timer to delay collection. When the delay is over, it begins collecting and closes the collection timer. According to the collected signals, the amplifier gain value for the next collection is calculated to realize amplifier gain auto control. To improve the data collection precision and the systems anti-jamming ability, multi-collection superposition algorithms are built into the collection system, which means reception waves of multi-excitation circles are averaged to eliminate random noise interference.

## 3. Key Technologies Adopted in the Excitation System

To obtain qualified LWD data, the crucial problem of the high noise confusion must be resolved [20]. In this paper, we propose three technologies to improve the signal-to-noise ratio: the dominant exciting wave bands of dipole source, the sine wave pulse excitation circuit, and the broadband impedance matching technique.

### 3.1. Dominant Exciting Wave Bands of Dipole Source

In recent times, dipole shear wave remote detection technologies have been significantly improved for traditional wireline logging, which can effectively detect fracture structures through boreholes and near-boreholes, identify the borehole’s hidden reservoir structure, and provide an important application for shale gas reservoir detection [21,22,23].

To reduce the complexity of the corresponding calculations, the underground layer was considered infinite, and the dipole shear wave source was regarded as a point source. Thereafter, aiming at practical judgement of dipole source technology for acoustic LWD and its dominant exciting wave bands, theoretical derivations were performed to obtain the remote-field displacement spectrum asymptotic expression of the SH and SV shear waves:

(1) {uφ ~ [iρβωD(ω,k0)sinθsinφ]eiωRβ4πρβ2RS(ω)uθ ~ [ρω2F(ω,k0)sinθcosφ]eiωRβ4πρβ2RS(ω)

The remote-field radiation directive property of the SH and SV shear waves are contained in expression 2:

(2) $$\{\begin{array}{c} R_{SH}\left(\omega ;\theta ,\varphi \right)=i\rho \beta \omega D\left(\omega ,k_{0}\right)sin\theta sin\varphi \\ R_{SV}\left(\omega ;\theta ,\varphi \right)=\rho \omega^{2}F\left(\omega ,k_{0}\right)sin\theta cos\varphi \end{array}$$

The calculation of amplitude coefficients D and F is the core question in expression 2. The amplitude coefficient matrix equation of the LWD dipole source inside and outside the borehole can be obtained, and then the amplitude coefficients D and F can be obtained by solving the matrix equation in [24]. Lastly, the remote-field radiation directive property under a random frequency can be calculated.

**Figure 4:**
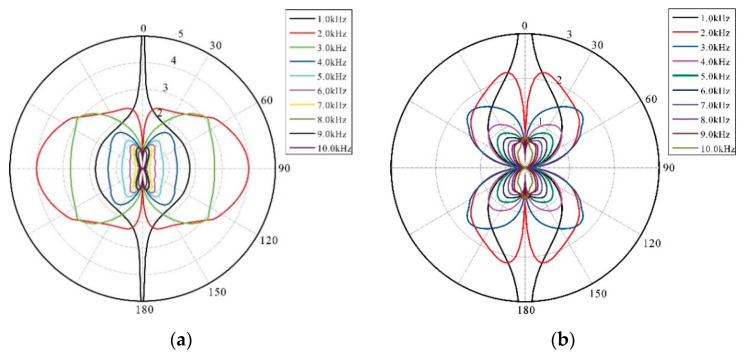
Far-field radiation patterns of the LWD SH (**a**) and SV (**b**) waves for different source frequencies.

**Figure 5:**
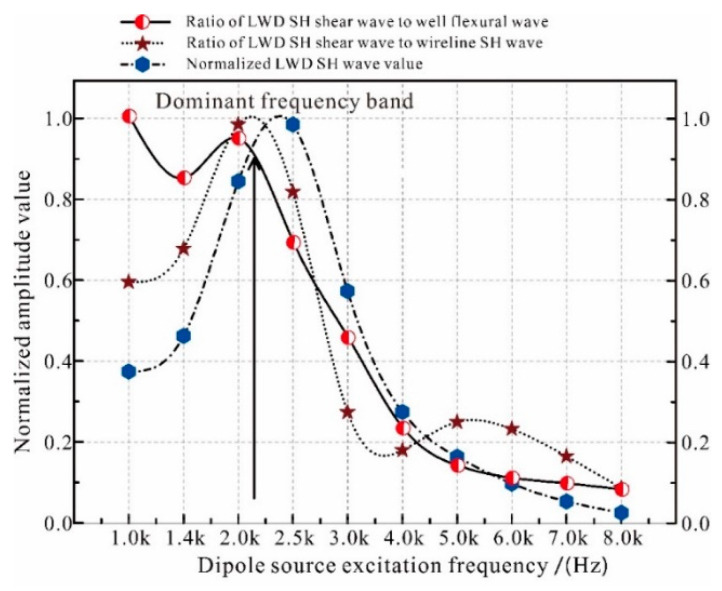
Relationships between source frequencies and LWD SH wave normalized amplitudes.

Far-field radiation patterns of the LWD SH and SV waves for 10 different source frequencies were calculated, and are shown in Figure 4. The relationships between source frequencies and the LWD SH wave normalized amplitudes were shown in Figure 5; it shows the amplitude ratio of the LWD SH shear wave and borehole flexible wave, the amplitude ratio of the LWD SH shear wave and open-hole SH shear wave, and the normalized amplitude of the LWD SH shear wave. These figures certify that the dominant frequency band for this model is between 1.8 and 2.5 kHz.

### 3.2. Sine Wave Pulse Excitation Circuit

To avoid the interference of the borehole flexural wave being excited outside of the dominant frequency band, and guarantee that the excited dipole acoustic energy is concentrated on the dominant frequency band, the transducer exciting signals must be controllable and obtain a narrow frequency band (such as multicycle sine waves) [25,26]. On the other hand, reducing the acoustic excitation frequency will cause it to be closer to the frequencies of the drilling noise and drilling fluid cycle noise. This should be addressed by improving the transmitting transducer excitation power [27]. Currently, most acoustic well logging transmitting transducers are fired by single-pulse excitation circuits, whose advantage is their simple structure and wide frequency band [28,29,30,31]. Meanwhile, the acoustic signal frequencies excited by these circuits is not adjustable, and the excited transducers only work at their resonance frequencies, no matter the impulse width of the excitation signals [32,33,34].

**Figure 6:**
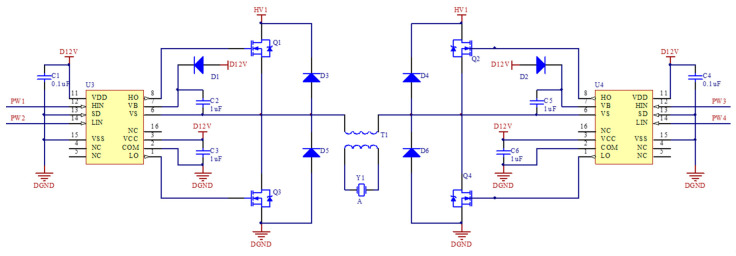
Core electronic structure of full bridge SPWM circuit.

**Figure 7:**
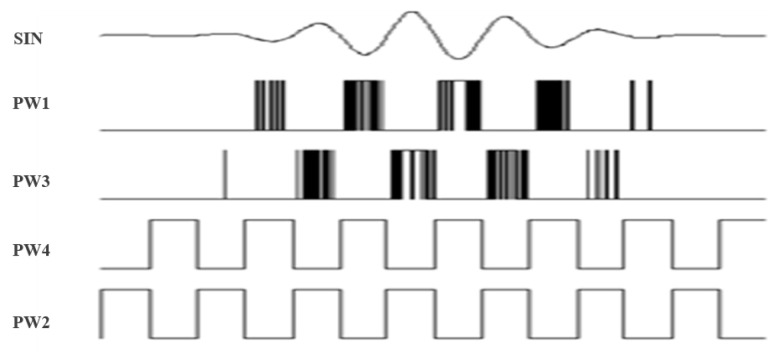
Working sequence chart of SPWM.

To solve this problem, we proposed a gate sine wave pulse excitation circuit based on a push–pull power amplification structure [35]. Sine wave sinusoidal pulse width modulation (SPWM) is a series of square pulses whose equal cycle and unequal duty ratio are changed as regularly as a sine wave. Figure 6 shows the core structure of the full bridge circuit. Q1–Q4 are four V-groove metal oxide semiconductors, Y1 is a transmitter transducer, T1 is an impedance transformer, and U3 and U4 are two full bridge driver chips. As shown in Figure 7, while producing the positive signal of a sine wave, Q2 and Q4 are two low frequency relative semiconductors, as Q2 is off and Q4 is on. Meanwhile, Q1 and Q3 are two high frequency complementary semiconductors, as Q1 is the SPWM pulse sequence and Q3 is off. Thereafter, positive sine wave signals are added to transformer T1 along the path of ‘Q1-T1-Q4’. Similarly, while producing the negative signal of a sine wave, PW3 supplies SWPM pulse sequences for Q2, Q3 supplies a low frequency conducting signal, and Q1 and Q4 are off. The working status goes around in circles, and whole sine wave impulse signals are produced.

**Figure 8:**
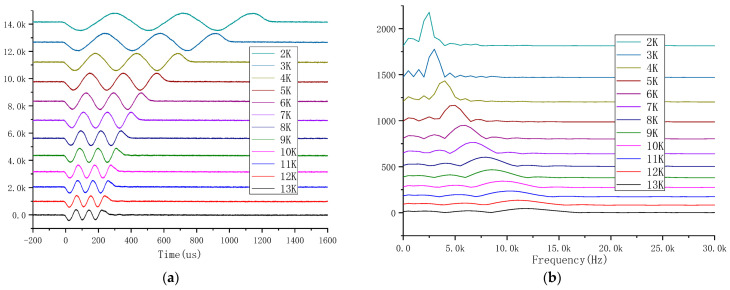
Time-domain (**a**) and spectrum waves (**b**) excited by SPWM excitation circuit.

After this, the SPWM signal excitation circuit was designed and processed. Experiments were conducted to excite one LWD transducer of the designed circuit. The excitation signals were recorded using the same high resolution oscilloscope, while excitation frequencies were changed from 2 KHz to 13 KHz and other parameters were kept the same. The time-domain figures and spectrum figures are shown in Figure 8. This circuit produced ideal three circle sine waves. The frequency character was improved and its power was larger as its excitation frequency decreased. This was because the excitation transducer is a dipole transducer, whose resonance frequency is about 2 KHz.

### 3.3. Broadband Impedance Matching

Acoustic transducers are used to realize energy transformations between alternating electric signals and sonic signals, and acoustic logging while drilling technology generates longitudinal monopole waves, dipole shear waves, and quadrupole waves by exciting the same four piezoelectric transducers of different frequencies and combination modes [6,36]. That is, the excitation circuit should produce at least three high-power signals of different frequencies. The electronic characteristics of piezoelectric transducers are always those of a capacitor or inductor, while the excitation source (excitation circuit and transformer) characteristics are those of a resistance. Connecting the transducer and excitation source directly will bring about an impedance mismatch, and the excitation power will reflect this significantly. The excitation efficiency of piezoelectric transducers is directly influenced by the electrical impedance matching performance of the transducers and excitation circuits; thereafter, impedance matching circuits are usually adopted [37]. On the other side, transducer electrical impedances clearly alter with the excitation frequency, whereas the excitation circuit impedances remain unchanged. As acoustic LWD technology can obtain monopole, dipole, and quadrupole waves with the same excitation circuit, a broadband impedance matching technique for LWD transducers and excitation circuits must be researched.

**Figure 9:**
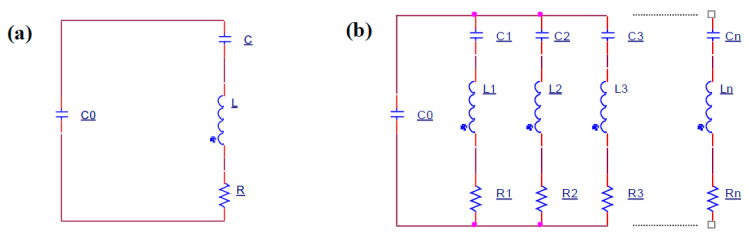
Equivalent circuit of piezoelectric transducer: (**a**) single mode equivalent circuit, (**b**) multimode equivalent circuit model.

Until now, impedance matching technologies have usually focused on single frequencies (mainly the resonance frequency), and the electronic circuit in Figure 9a was used to simulate transducer impedance characteristics, whereas the impedance characteristics can be expressed by Equation (3). However, while researching transducers working at multi frequencies, the simulation of the electronic circuit should be optimized as in Figure 9b, and the impedance characteristics should be changed to Equation (4).

(3) $$Y=\frac{j\omega^{2}CRC_{0}-\left(\omega C_{0}\right)\left(\omega^{2}LC-1\right)+\omega C}{\omega RC+j\left(\omega^{2}LC-1\right)}$$

where ω stands for angular frequency, and ω=2πf

(4) $$Y=j\omega C_{0}+\overset{n}{\underset{i=1}{\sum }}{\left(R_{i}+j\omega L_{i}+\frac{1}{j\omega C_{i}}\right)}^{-1}$$

where n stands for the number of resonance points.

Based on the above theories, the Smith Chart module of advanced design system (ADS) was utilized to design the impedance matching network. As demanded by LWD technology, the working frequency bands were set as 2~12 kHz, and the dominant frequency was set as 7 kHz. The transducer impedance data at 7 kHz can be set as the objective target to match the signal source impedance, and the matching impedance network can be obtained by ADS. Thereafter, the impedance network was added to ADS, and then the impedance characters of 2~12 kHz were optimized based on a genetic algorithm. Lastly, the effect of the matching network could be evaluated by observing the transducer active power before and after impedance matching by circuit simulation testing.

**Figure 10:**
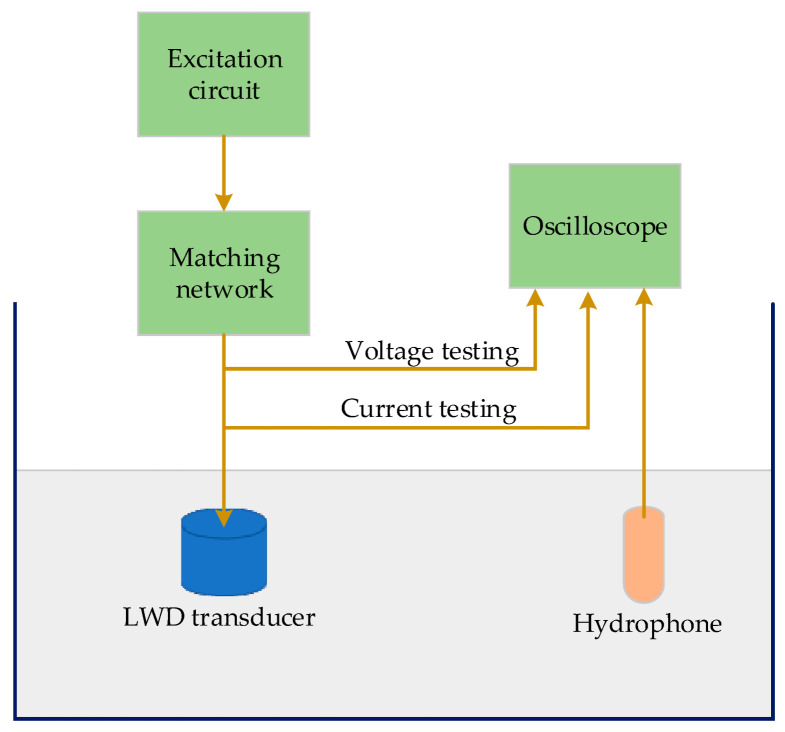
Schematic representation of the structure of impedance matching experiments.

Impedance matching experiments were conducted to test the matching network effectivity (Figure 10). To reduce uncontrollable influencing factors, the transducer excitation circuit was designed to work with or without matching networks. In the process of the experiments, the same LWD transducer was first excited by the circuit without matching networks, then the excitation signals were collected while the excitation frequencies were changed from 2 KHz to 12 KHz and the other parameters were kept unchanged. Thereafter, the same experiments were conducted, while the circuit was with the matching networks. Table 1 shows the active power of the transducer tested before and after impedance matching. It shows that the average active power was improved significantly. It can also be concluded that the transducer achieved the best power at the resonance frequency before impedance matching, and it realized the ideal power at the demanding frequencies after matching.

## 4. Key Technologies Adopted in Reception System

**Figure 11:**
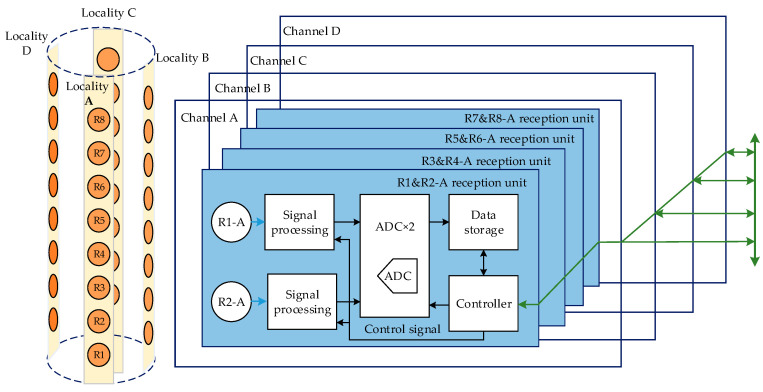
Schematic representation structure of reception system.

Circular slice piezoelectric transducers were adopted in the reception system. Four transducers were built up as one receptor, and arranged along the tool’s circumferential orientation at 90° intervals. There were eight such receptors arranged along the tool’s axial orientation in all. Thereafter, we encapsulated eight transducers of the same azimuth as one data collection channel, and signals from every two adjacent transducers in the same channel were processed by one electronic unit. That is, the reception system had sixteen of the exact same reception electronic units, and each unit only had a physical address. Thereafter, every electronic unit communicated with the upper major control system only according to its address, by the CAN bus (Figure 11).

**Figure 12:**
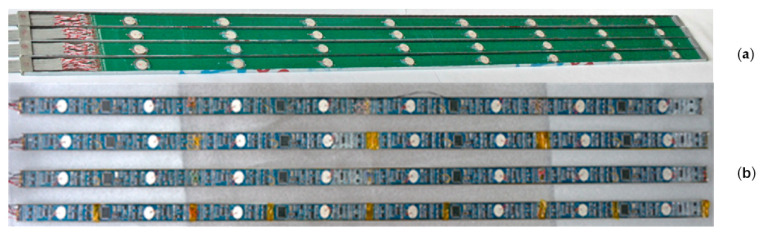
Traditional reception circuits (**a**), and intellectualized active reception circuits (**b**).

The aforementioned 16 electronic units must begin collecting at the same time, and traditional reception technology needs a large amount of wiring. This structure separates transducers and their processing circuits, which is beneficial to guarantee the collecting synchronism, but it is easy for crosstalk and outside disturbances to occur. Meanwhile, the junction quantity of the confined units may restrict the transducer quantity when connected in this way [38]. Thereafter, we proposed the technology of the intellectualized active reception transducer, which involves integration envelopment of the reception electronic unit and reception transducer. This structure obviously reduces the amount of junction wire, and can decrease crosstalk and outside disturbances (Figure 12).

**Figure 13:**
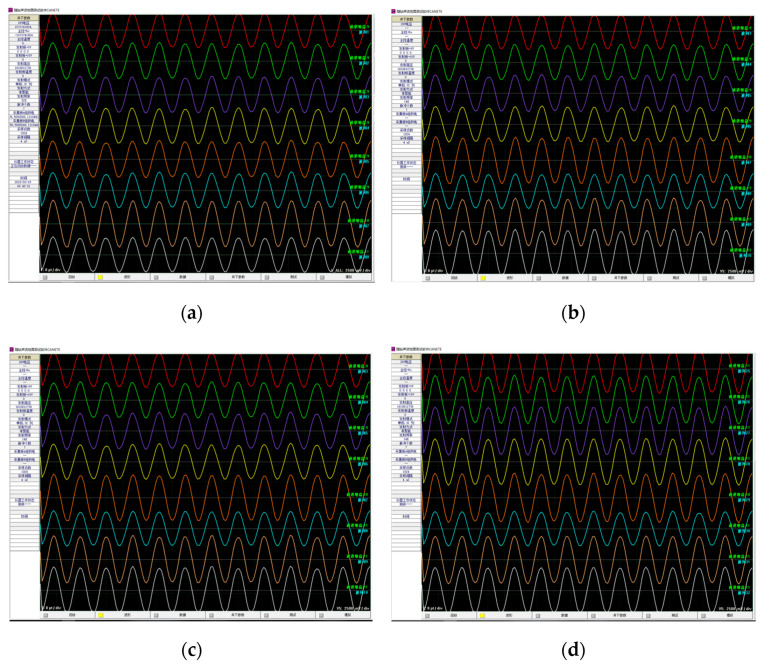
Test waveforms of axial transducer data trace. (**a**–**d**) stands for orientation A, B, C, and D, respectively.

**Figure 14:**
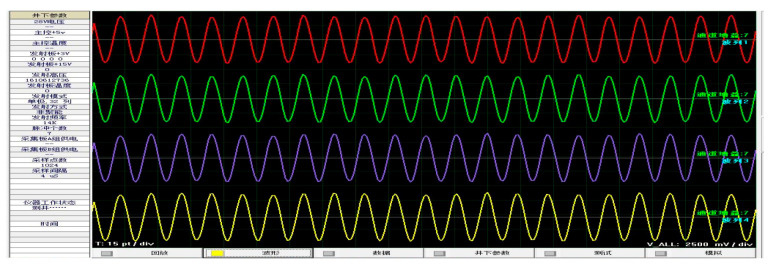
Test waveforms of the data track of the circumferential transducer.

The consistency of parameters between these 32 reception transducers is extremely important because they can determine a combined effort directly [39]. To select 32 reception transducers of certain consistency, the impedance characteristics of hundreds of transducers were measured. Thereafter, the probability statistics of the impedance endings were carried out to obtain their expected value [40]. Lastly, the 32 transducers were selected according to their variance yields. Meanwhile, the 32 reception channels of the 16 reception units must utilize a good consistency of amplifier gain and filter bandwidth. Hence, we measured them using electronic experiments: the same standard sine signal was set as the transducer signal and obtained access to the 32 reception channels, then the LWD tool began to work and obtained 32 channels of processed data in real time. Figure 13 shows the eight reception data curves of the same circumferential orientation, and Figure 14 shows the eight reception data curves of the same axial orientation. The amplitude of the sine signal was 200 mV, and the amplifier gains were 21 dB. We can conclude that their biggest phase difference was 2.6% and their biggest amplitude difference was 9.45%.

## 5. Experiments of the Acoustic LWD Tool

**Figure 15:**
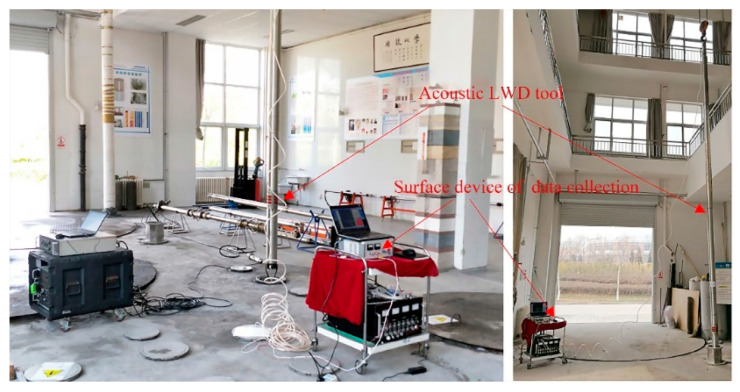
Acoustic LWD testing in room well group.

Experiments were performed in our azimuthal anisotropy module well, as in Figure 15, the LWD acoustic logging tool was connected with ground data acquisition equipment using cables of 40 m; this tool could work in a wireline logging module or LWD logging module [41,42,43].

The hole size of the azimuthal anisotropy module well was 0.245 m, and its depth was 10 m. The upper 5 m were toroidal anisotropy layers (the direction of faster waves was north–south. The layer velocity of the longitudinal wave was 4750 m/s and the shear wave was 2500 m/s, while the direction of the slower wave was east–west, and the layer velocity of the longitudinal wave was 3100 m/s, while the shear wave was 19,500 m/s. The lower 5 m were uniform layers, whose longitudinal wave velocity was 3250 m/s and shear wave was 1900 m/s.

**Figure 16:**
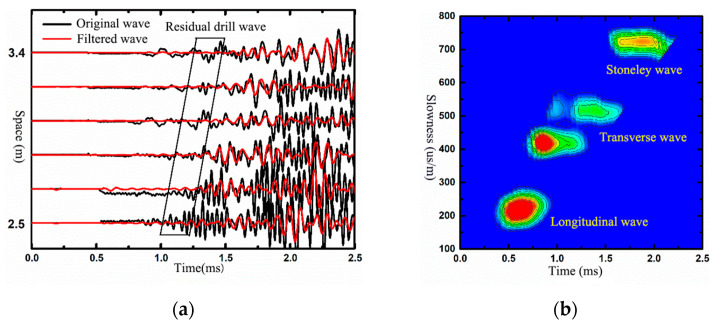

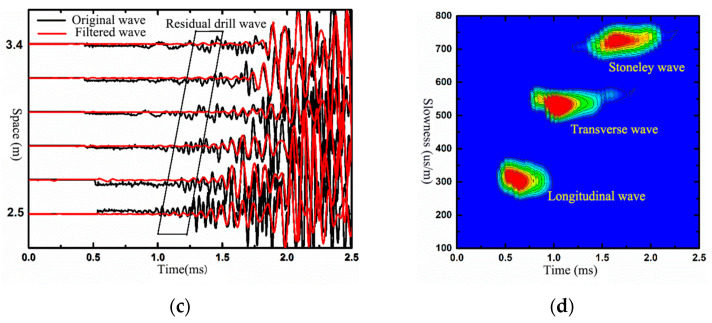
Waveforms and STC results of a depth point in anisotropic formation: (**a**,**b**) is in upper module well; (**c**,**d**) is in lower module well.

Figure 16a shows the original wave and filtered wave (9~15 kHz) of the upper module well measured at a single point. The reception direction was north–south. The wave data, processed by slowness-time coherence technology (STC), are shown in Figure 16b. It is shown that the bandpass filter can depress the impact of the drill wave. The formation longitudinal wave, transverse wave, and Stoneley wave can be distinguished clearly, and their corresponding slowness established as 215 μs/m, 410 μs/m, and 720 μs/m. Adopting the same process method to the lower module well, the slowness of the longitudinal wave, transverse wave, and Stoneley wave was 303 μs/m, 520 μs/m, and 714 μs/m.

These two series of formation velocities coincided with the module well parameters (as shown in Table 2). That is to say, this tool obtained real formation signals.

To certify the measure flexibility and signal accuracy of this logging tool, it was tested in the azimuthal anisotropy module well while lifting up and falling down. In the process of testing, the tool slowly fell down from the upper toroidal anisotropy layers to the lower uniform layers, keeping a still measurement for 2 s, and then lifting the tool up to the upper layers, keeping a still measurement for 2 s.

**Figure 17:**
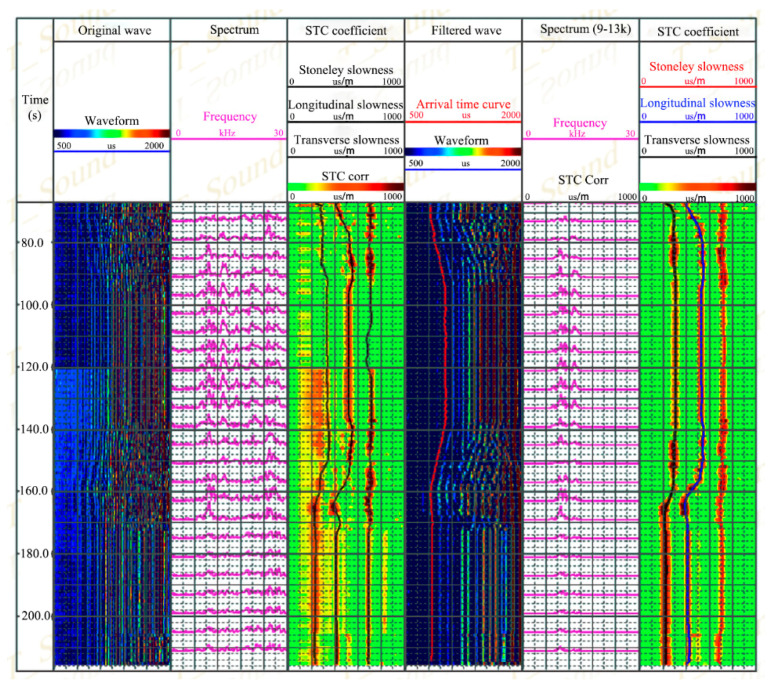
Continuous acquisition and STC results of anisotropic formation.

Figure 17 shows the original logging data and the processing ends. It can be concluded that the signal-to-noise ratio of the filtered waves showed obvious improvement compared with the original wave. Slowness-time coherence processes are utilized for the original (row 4) and filtered wave (row 7), and the slowness of the longitudinal wave, transverse wave, and Stoneley wave could be obtained for both waves. Meanwhile, the signal-to-noise ratio of the filtered wave was much better than that of the original wave. While the maintained stillness, and the slowness of the longitudinal, transverse, and Stoneley wave remained consistent, that is, the tool had obvious stability.

To test the LWD acoustic tool in complicated drilling circumstances, it was tested in a calibration well and normal well, working in the module of the cable, sliding eye, and logging while drilling.

**Figure 18:**
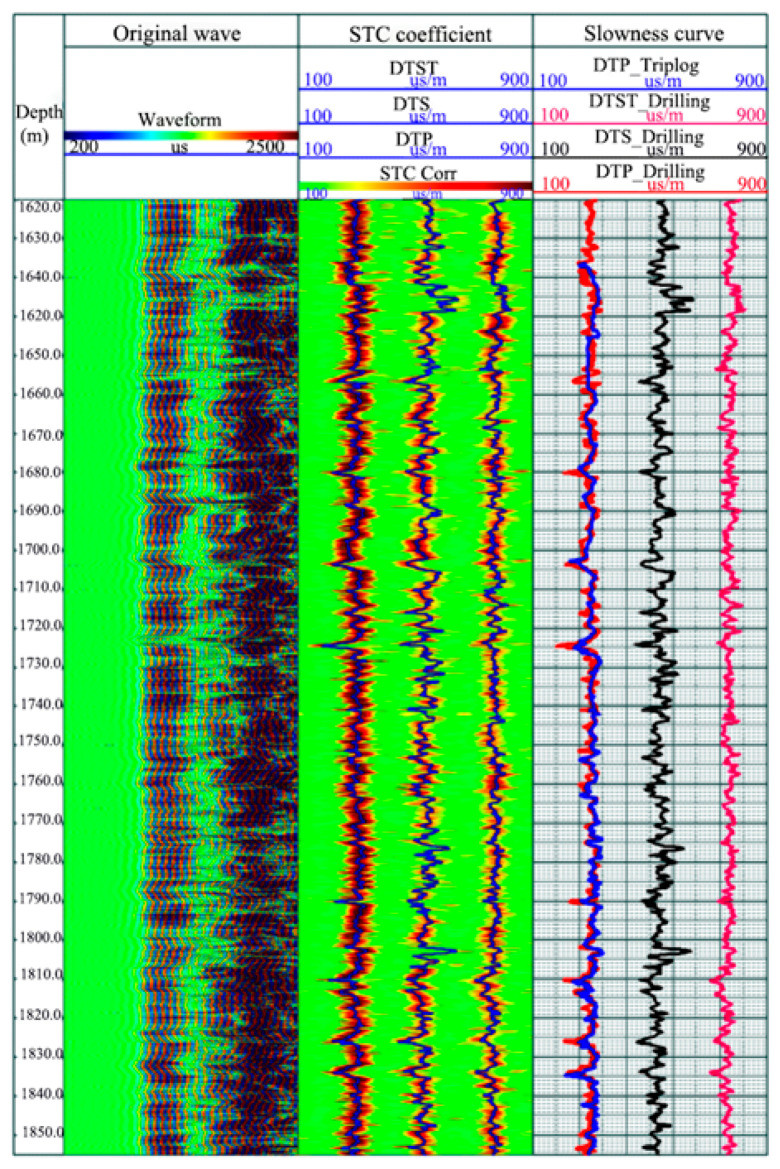
Comparison of LWD and wireline logging results in the X-2 well.

To obtain the operational performance in the module of logging while drilling, the tool performed LWD measurements in the X-2 well. Shown in Figure 18, row 1 is the curve of depth; row 2 is the variable density figure of the original logging waves; row 3 is the process ending of the slowness-time coherence method; and row 4 is the formation longitudinal, transverse, and Stoneley wave, where red and blue curves represent the longitudinal time difference curve of the LWD and sliding eye modes. It can be concluded that the end of the LWD and sliding eye modes achieved significant consistency, meaning the tool has good reliability and stability.

**Figure 19:**
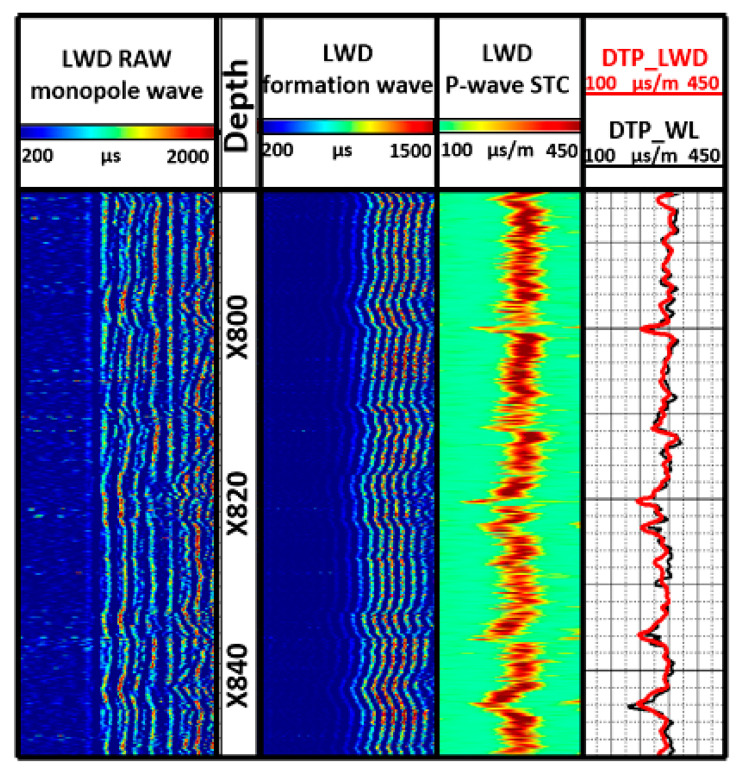
Comparison of LWD and wireline logging results in X-3 well.

Figure 19 shows the LWD original wave and their process endings. Row 1 is the LWD original wave stored underground, where the formation signal can be observed clearly, and the disturbance of the drill wave was also exposed. Row 2 shows the filtered LWD wave data. Row 3 shows the longitudinal time difference wave, obtained by velocity corresponding analysis of row 2, and which has significant signal quality and correlation characteristics. Row 4 (red color) shows the formation longitudinal time difference wave extracted from the coherence figure. To certify the reliability of the LWD formation longitudinal time difference, Row 4 (black color) also shows the formation longitudinal time difference measured by wireline logging, which proves that the time difference waves coincide with each other, and that this tool can obtain high quality formation acoustic signals and time difference data.

## 6. Discussion and Conclusions

To obtain qualified LWD data, a new acoustic LWD tool was researched in this paper. First, the overall design, including the physical construction, electronic system, and flowchart, was introduced. Then, the practicality judgement of the dipole shear wave remote detection technology on the acoustic LWD was carried out, and its dominant exciting wave bands were certified to be between 1.8 and 2.5 kHz. Thereafter, a gate sine wave pulse excitation circuit based on a push–pull power amplification structure was designed, and experiments showed that this circuit could utilize the ideal sine waves of demanding frequencies. After that, broadband impedance matching technologies were adopted to realize high-efficiency monopole, dipole, and quadrupole wave excitation by the same circuit, and experiments showed that the active power after matching was much bigger than that before matching. The intellectualized active reception circuit was designed to improve the signal-to-noise ratio, and good parameter consistencies for the 32 reception transmitters were achieved. Lastly, the whole tool was tested in the azimuthal anisotropy module well, calibration well, and normal well, working in the modules of the cable, sliding eye, and logging while drilling. The experiments showed that this tool obtained qualified data.

Compared with traditional tools, this paper designed and adopted several technologies to improve the signal-to-noise ratio and detection range. This is of significance to oil and gas exploration, and other corresponding fields.

## Figures and Tables

**Table 1:** The measured active power in the preset frequency band of the transducer before and after impedance matching.

Frequency (kHz)	Active Power before Matching (W)	Active Power after Matching (W)	Normalized Power before Matching	Normalized Power after Matching
2	0.16	9.67	0.21	0.96
3	0.21	8.30	0.27	0.82
4	0.13	4.56	0.17	0.45
5	0.35	3.60	0.45	0.36
6	0.46	5.78	0.59	0.57
7	0.78	7.26	1.00	0.72
8	0.62	5.31	0.80	0.52
9	0.31	2.69	0.40	0.27
10	0.40	6.13	0.51	0.61
11	0.24	7.89	0.31	0.78
12	0.29	10.12	0.37	1.00

**Table 2:** Comparison table of standard formation velocity and measured velocity.

Item	Standard Velocity (m/s)	Measured Slowness (μs/m)	Measured Velocity (m/s)
Upper north–south longitude wave	4750	215	4651
Upper north–south transverse wave	2500	410	2439
Upper north–south Stoneley wave	/	720	1388
Lower longitude wave	3250	303	3300
Lower transverse wave	1900	520	1923
Lower Stoneley wave	/	714	1400
